# Facial Feedback Affects Perceived Intensity but Not Quality of Emotional Expressions

**DOI:** 10.3390/brainsci5030357

**Published:** 2015-08-26

**Authors:** Janek S. Lobmaier, Martin H. Fischer

**Affiliations:** 1 Institute of Psychology, University of Bern, Fabrikstrasse 8, 3012 Bern, Switzerland; 2 Division of Cognitive Sciences, University of Potsdam, Karl-Liebknecht Str. 24/25, House 14, 14476 Potsdam-Golm, Germany; E-Mail: martinf@uni-potsdam.de

**Keywords:** embodied cognition, emotional expression, emotion recognition, facial feedback, face morphing

## Abstract

Motivated by conflicting evidence in the literature, we re-assessed the role of facial feedback when detecting quantitative or qualitative changes in others’ emotional expressions. Fifty-three healthy adults observed self-paced morph sequences where the emotional facial expression either changed quantitatively (*i.e.*, sad-to-neutral, neutral-to-sad, happy-to-neutral, neutral-to-happy) or qualitatively (*i.e.* from sad to happy, or from happy to sad). Observers held a pen in their own mouth to induce smiling or frowning during the detection task. When morph sequences started or ended with neutral expressions we replicated a congruency effect: Happiness was perceived longer and sooner while smiling; sadness was perceived longer and sooner while frowning. Interestingly, no such congruency effects occurred for transitions between emotional expressions. These results suggest that facial feedback is especially useful when evaluating the intensity of a facial expression, but less so when we have to recognize which emotion our counterpart is expressing.

## 1. Introduction

Efficient recognition of emotional expressions is fundamental when interpreting and predicting other people’s behaviour e.g., [1]. From the expression on a person’s face we can infer how they feel, which is an important pre-requisite for empathy. Moreover, we seem to automatically and non-consciously mimic the facial expression of an interaction partner e.g., [2,3]. Embodied cognition theories suggest that an internal simulation of a perceived emotion through facial mimicry facilitates the recognition of this emotion. Specifically, individuals might detect changes in emotional expressions of another person not only visually but also through the feedback from their own facial expressions [4,5,6,7]. Based on such observations it has been proposed that facial mimicry is an important source of feedback involved in the recognition of facial expressions [8,9,10,11]. On the other hand, work with patients suffering from congenital facial paralysis suggests that activation of facial muscles in the observer might not be a necessary component of emotion recognition in other people’s faces [12]. Here we set out to scrutinize the influence of facial feedback on the perception of expressions on other people’s faces.

In a study apparently supporting the idea that facial feedback is involved in the recognition of facial expressions, Niedenthal *et al*. [5] investigated whether observer mood influences the interpretation of seen emotional expressions. They first induced in their participants either a happy, sad, or neutral emotional state with movie clips and music. Then participants saw morph sequences of happy or sad faces turning into faces with a neutral expression. Each morph sequence consisted of 100 frames and participants used a slide bar control to stop the sequence where they no longer saw the initial facial expression. The authors found that facial expressions that were congruent with the emotion induced in the participant were perceived to persist longer than emotion-incongruent expressions. Specifically, happy participants perceived a happy expression in the happy-to-neutral sequences to last longer than sadness in the sad-to-neutral sequences and sad perceivers saw sad expressions to last longer in the sad-to-neutral movies than in the happy-to-neutral sequences. While these emotion-congruency effects found by Niedenthal *et al*. [5] seem to indicate an effect of observer mood on visual emotion perception, they can also be explained in terms of facial mimicry: happy participants might have mimicked happy expressions in the happy-to-neutral clips more efficiently and for a longer time than sad participants, thereby biasing their responses via facial proprioception. By the same token, sad participants might have mimicked sad expressions more efficiently and for longer than happy participants. In the absence of explicit proprioceptive manipulations it remained unclear whether the observed bias was visually or mimicry based.

In a follow-up study, Niedenthal *et al*. [4] again induced happy, sad, or neutral emotional states by means of movie clips and music, but this time participants saw morph sequences of emotional expressions turn into categorically different expressions. Specifically, happy and sad expressions of the same individuals were digitally blended, producing 100-frame movies in which a sad expression gradually turned into a happy expression, and *vice versa*. Again participants were requested to adjust a slide bar to indicate where they stopped seeing the initial expression. This time the authors found that happy participants saw the offset of happiness earlier than did the sad participants; and that sad participants saw the offset of sadness earlier than the happy participants—A result opposite to the earlier congruency effect.

We note that these mismatching results could be attributed to a number of methodological factors, foremost among which figures the structure of the morph sequences which now consisted of blends between two separate emotional categories instead of a blend between one emotional category and an emotionally neutral state. In any event, the pattern of results did not allow adjudicating between a visual and a proprioceptive account of biases in emotion perception.

In a third experiment with the same cross-category morph sequences, Niedenthal *et al*. [4] tried to clarify whether or not facial mimicry might be involved in the observed effects. In this experiment mood was not manipulated; instead, half the participants were asked to hold a pen in their mouth using both their teeth and their lips, an intervention known to disrupt facial mimicry [13]. The authors expected that participants who could freely mimic emotional expressions would see the facial expressions changing earlier than participants who were not able to mimic the emotions due to the pen in their mouth. Indeed, this is what the authors found: individuals who could freely mimic the faces noticed the changes in emotion earlier than individuals with restricted mimicry, presumably because the former could rely on situation-congruent facial feedback.

Despite these systematic results in favor of facial feedback through mimicry, a methodological problem exists with this third experiment. Specifically, mimicry was restricted by asking participants to hold a pen “using both their teeth and their lips”. The original study by Strack *et al.* [13], however, differentiated between these two ways of holding the pen in the mouth: by holding the pen between the teeth, participants are forced to activate the zygomaticus major, a muscle that is important for smiling. On the other hand, holding the pen between the lips makes smiling impossible and instead activates frowning-related muscles. Hence, it is unclear to what extent facial feedback was restricted in the critical experiment of Niedenthal *et al.* [4]. The lack of specificity of their suppression effect suggests that the authors succeeded in suppressing both happy and sad mimicry. However, this was also a missed opportunity to test the prediction of emotion-specific interference through the pen manipulation and to assess the presence and direction of emotion-specific congruity effects that is predicted by embodied cognition theory. In the present study we blocked free facial mimicry by restricting facial feedback to either a frowning or smiling expression and measured the consequences for emotional recognition of congruent and incongruent facial expressions. The present study was thus designed to further investigate the role of facial feedback in emotion recognition and to further qualify the findings of previous studies e.g., [4,5].

To summarize, two methodological mix ups in previous work need to be disentangled to clarify the role of facial feedback in emotion perception. First, both the appearance and the disappearance of emotional facial expressions should be investigated to assess the relevance of congruent *vs.* incongruent visual evidence. Secondly, two different types of facial expressions should be clearly manipulated in observers to assess the presence and direction of any congruity effects. The present study sets out to do this in order to clarify the role of facial feedback in the perception of facial expressions in others.

Similar to Niedenthal *et al.* [4,5] we also created facial morph sequences but we included all possible transitions between happy and neutral, sad and neutral, neutral and happy, neutral and sad, happy and sad, and sad and happy faces, respectively. Moreover, we distinguished three facial suppression conditions: no pen, pen between lips, and pen between teeth. This augmented design allowed us to test the following specific predictions: according to embodied cognition theory, participants should see happiness longer in the happy-to-neutral morph sequences and they should see happiness earlier in neutral-to-happy sequences when they are forced to smile (pen between teeth condition) compared to when they are prevented from smiling (pen between lips condition). Conversely, participants who are prevented from smiling are expected to see sadness longer in the sadness-to-neutral condition and earlier in the in the neutral-to-sadness condition than participants who are forced to smile. In morph sequences where the emotional expressions change categorically (*i.e.* happy-to-sad and sad-to-happy) two possible outcomes are conceivable. Either happiness is perceived longer and detected earlier when participants are forced to smile, compared to when they are prevented from smiling (congruency effect). Alternatively, participants might see a qualitative change from happiness to sadness earlier if they are forced to smile and a change from sadness to happiness earlier when they are prevented from smiling.

In the sequences where sad or happy expressions turn into neutral expressions and *vice versa*, the emotion remains the same albeit getting weaker or stronger. Congruent facial feedback may then facilitate the perception of the congruent emotion. When an expression changes qualitatively, incongruence between the proprioceptively experienced emotion and the seen emotion may be detected earlier, simply because the experienced expression is in conflict with the seen emotion.

## 2. Experimental Section

### 2.1 Participants

Fifty-nine undergraduate students (39 women and 20 men) from the University of Dundee (Scotland), aged between 18 and 25 years, volunteered to take part in this study. All participants reported normal or corrected to normal visual acuity. All subjects gave their written informed consent for inclusion before they participated in the study. The study was conducted in accordance with the guidelines of the British Psychological Society and with the Declaration of Helsinki. The written informed consent included participants’ right to terminate participation at any time without penalty. Eventually, data of six participants had to be excluded from analyses because of incomplete data sets (*i.e.*, participants who had five or more missing responses in one block).

### 2.2 Apparatus

The study was run on a PC using SuperLab 4.0^®^. The stimuli were presented on a 21 computer screen with a resolution of 1280 × 1024 pixels and a color depth of 24 bits. The screen was placed approximately 50 cm away from the observer. The stimuli appeared in the center of the screen with a width of approximately 15 cm, subtending a visual angle of approximately 5.72 deg horizontally. A standard plastic ballpoint pen was used to induce facial muscle activation.

### 2.3 Stimuli

The visual stimuli were created from four face identities (two men and two women), each expressing either a neutral, happy, or sad expression in separate photographs, provided by the Karolinska face data-base [14] (the identities af01, af20, am02, and am11 were used). These expressions were rated by an independent sample consisting of 10 men and 10 women for clearness and intensity. Each emotional face was presented together with five emotional labels (sad, angry, happy, fearful, and neutral) and participants were asked to indicate which of these emotions best described the expressed emotion. Additionally, participants indicated how intense they thought this emotional expression was on an analog scale ranging from 1 (not at all) to 30 (very intense). All emotional expressions were recognized with high accuracy: happiness was never misclassified; neutrality and sadness were correctly identified in more than 79% of the cases (for similar results see [15]). The intensity ratings of sad and happy emotions did not differ significantly, *t* (19) = 0.81 *p* = 0.428. For each of these identities, six morph sequences were created using Psychomorph computer graphics software [16]. Morph sequences were created between the expressional faces and their neutral counterparts (happy to neutral, sad to neutral, neutral to happy, and neutral to sad) and between the two emotional expressions (sad to happy, happy to sad), each containing 40 steps. Stimuli can be provided on request.

### 2.4 Task and Procedure

Participants were seated in front of the computer screen on which the morph sequences were presented and responded using the computer keyboard. They received oral and written instructions and were given three practice trials before the experiment proper started. The experiment consisted of three blocks, each containing 24 trials (four identities, six morph sequences). Participants were told that two of these blocks would require them to hold a pencil in their mouth, and that the experimenter will explain this as needed. A trial started with the presentation of a face showing one of three expressions (neutral, happy, or sad). By pressing the space bar, participants displayed the next step in the morphing sequence, thus gradually changing the facial expression. The task was to indicate at which step of the morphing sequence they perceived a change in the initial expression by pressing the “J” key instead of the space bar. The frame number at which the changed expression was perceived was recorded by the computer which then initiated the next trial.

Participants each underwent three different conditions. In all three conditions the task and procedure was the same, except for one detail: In one block they were asked to hold a pen between their teeth (without the lips touching the pen), in a second block they were required to hold the pen using their lips. The experimenter individually demonstrated how participants were to hold the pen. Importantly, in both conditions the pen pointed forward, as in Strack *et al*. (1988, Figure 1). Finally, in the third block participants completed the tasks without a pen in their mouths. The order of blocks was counterbalanced across participants.

### 2.5 Data analysis

The critical frame numbers at which the change of expression was perceived were averaged across stimulus identities, separately for each morph sequence and participant, for further analyses. The results of the morph sequences containing a neutral expression were analyzed separately from the results of the morph sequences in which one expression turned into a qualitatively different one (sad-happy). This was done because the amount of facial changes between consecutive frames was not comparable between these two types of morph sequences, and because in the sequences containing a neutral expression there was only a quantitative difference between the two extremes, whereas in the transitions between happy and sad facial expressions there was a qualitative change between the initial and final emotion. We calculated a 4 × 3 × 2 repeated measures Analysis of Variance (ANOVA) on the critical frame numbers from happy-neutral and sad-neutral sequences, with morph sequence (happy-neutral, neutral-happy, sad-neutral, neutral-sad) and pen condition (induce smile, induce frown, no induction) as within participant factors. Because a number of studies have reported a female advantage in recognizing emotional expressions e.g., [17,18,19,20] we added participant sex as a between participant factor. On the critical frame numbers from morph sequences from happy to sad and *vice versa*, a 2 × 3 × 2 repeated measure ANOVA was calculated, with morph sequence (happy-sad, sad-happy) and pen condition (induce smile, induce frown, no induction) as within participant factors and participant sex as between participant factor.

## 3. Results

### 3.1 Expressions changing between happy/sad and neutral

The repeated measures 4 (morph sequence) × 3 (pen condition) × 2 (participant sex) mixed ANOVA revealed a significant main effect of morph sequence, *F* (3, 153) = 113.33, *p* < 0.001, *η_p_*^2^ = 0.69. There was no significant main effect of pen condition, *F* (2, 102) = 0.008, *p* = 0.992, *η_p_*^2^ = 0.00. But importantly, the interaction between morph sequence and pen condition reached statistical significance, *F* (6, 306) = 2.855, *p* = 0.01, *η_p_*^2^ = 0.053. There was no significant main effect of participant sex (*p* = 0.287) and no significant interaction including the factor participant sex (all *p*’s > 0.114).

The main effect of morph sequence indicates that the change of expression was detected fastest in the sequence neutral-to-happy (*M* = 16.499, *SD* = 0.82) followed by neutral-to-sad (*M* = 22.676, *SD* = 0.65), happy-to-neutral (*M* = 26.759, *SD* = 0.81) and sad-to-neutral (*M* = 27.292, *SD* = 0.57). Pair-wise comparisons (Bonferroni corrected) revealed significant differences between all morph-sequences (all *p*’s < 0.001), except for the comparison between happy-to-neutral and sad-to-neutral (*p* = 1.00).

**Figure 1:**
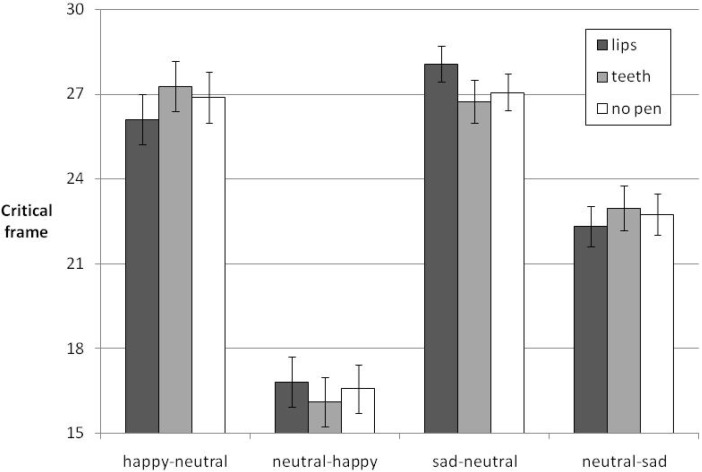
Means of critical frames averaged across participants, separately for the four morph sequences and three pen conditions, for expressions changing between happy/sad and neutral. Error bars depict SEMs.

The interaction between morph sequence and pen condition (the pen condition X morph sequence interaction remains significant when separately analyzing morph sequences including happy and sad expressions (happy: *F* = 6.345, *p* = 0.015, *η_p_^2^* = 0.109, sad: *F* = 5.276, *p* = 0.026, *η_p_^2^* = 0.092)) is explained by the predicted congruency effects. Specifically, in the morph sequence happy-to-neutral happiness was perceived to persist longest in the pen condition “teeth” (*M* = 27.28, *SE* = 0.88) followed by the no-pen condition (*M* = 26.89, *SE* = 0.90) and the “lips” condition (*M* = 26.11, *SE* = 0.89). Similarly, in the morph sequence neutral-to-happy happiness was perceived earliest in the “teeth” condition (*M* = 16.11, *SE* = 0.86) followed by the “no-pen” condition (*M* = 16.57, *SE* = 0.87) and the “lips” condition (*M* = 16.81, *SE* = 0.89). In the morph sequence sad-to-neutral sadness was perceived to persist longest in the pen condition “lips” (*M* = 28.07, *SE* = 0.62) followed by the no-pen condition (*M* = 27.06, *SE* = 0.64) and the “teeth” condition (*M* = 26.74, *SE* = 0.77). In the morph sequence neutral-to-sad sadness was perceived earliest in the “lips” condition (*M* = 22.31, *SE* = 0.72), followed by the no-pen condition (*M* = 22.75, *SE* = 0.73) and the “teeth” condition (*M* = 22.97, *SE* = 0.78) (see Figure 1). This is precisely the ordering of means as predicted by embodied cognition theory: Emotion-congruent pen manipulations bias observers towards perceiving that emotion. Post-hoc simple effects tests (one-tailed) were conducted to secure this predicted pattern statistically. These analyses confirmed that holding a pen between the teeth (smiling) compared to between the lips (frowning) delayed detection of the neutral face in the morph sequence happy-to-neutral, *t* (52) = 2.20, *p* < 0.02. The corresponding bias for earlier detection of happiness when initially observing a neutral face, however, yielded a non-reliable result, *t* (52) = 0.97, *p* < 0.17. In the morph sequences including sad faces, the pen condition had only a marginally reliable influence on the perception of on- or offset of sadness (for sad-to-neutral: lip > teeth condition, *t* (52) = 1.58 *p* < 0.07, for neutral-to-sad: lip < teeth condition, *t* (52) = 125, *p* < 0.11).

### 3.2 Expressions changing between happy and sad

A repeated measures 2 (morph sequence) × 3 (pen condition) × 2 (participant sex) mixed ANOVA revealed a significant main effect of morph sequence, *F* (1, 51) = 6.91, *p* = 0.011, *η_p_^2^* = 0.12. There was no significant effect of pen condition, *F* (2, 102) = 0.203, *p* = 0.82, *η_p_^2^* = 0.00 and no significant interaction between morph sequence and pen condition, *F* (2, 102) = 0.26, *p* = 0.77, *η_p_^2^* = 0.01. There was no significant main effect of participant sex (*p* = 0.308) but a significant interaction between morph sequence and participant sex *F* (1, 51) = 4.95, *p* = 0.03, *η_p_^2^* = 0.09.

**Figure 2:**
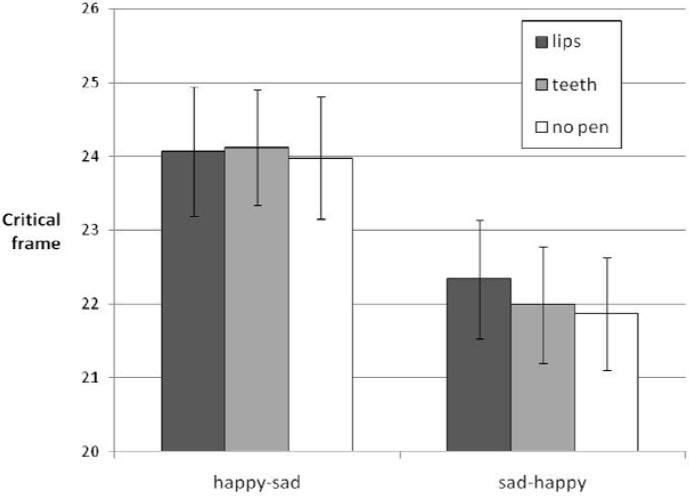
Means of critical frames averaged across participants, separately for the two morph sequences and three pen conditions, for expressions changing between happy and sad. Error bars depict SEMs.

The main effect of morph sequence indicates that the change of expression was detected earlier in the sequence sad-to-happy (*M* = 22.07, *SE* = 0.72) than in the sequence happy-to-sad (*M* = 24.06, *SE* = 0.77). The interaction between morph sequence and participant sex is explained by the fact that men detected the change of expression earlier in the morph sequence sad-to-happy (*M* = 20.56, *SE* = 1.21) than in the happy-to-sad sequence (*M* = 24.24, *SE* = 1.28), while women detected the change at about the same time (happy-to-sad: *M* = 23.87, *SE* = 84; sad-to-happy: *M* = 23.57, *SE* = 0.79) (see Figure 2).

## 4. Discussion

In this study we revisited the idea that facial feedback contributes to the perception of emotional expression in others’ faces. We followed up on previous evidence suggesting an influence of facial mimicry on emotion perception (Niedenthal *et al.*, 2000, 2001 [4,5]), and improved previous designs in two ways. First, we presented morph sequences with both transitions to/from emotional expressions and transitions to/from neutral states. Secondly, we selectively activated the use of smile- and frown-related facial muscles in our observers. We employed the paradigm introduced by Strack *et al.* [13], in which participants hold a pen either between their teeth or with their lips in order to activate certain facial muscles [21]: Holding the pen between the teeth is known to result in activation of the zygomaticus major (a muscle that is responsible for smiling); conversely, holding the pen with the lips activates the corrugator supercilii (a muscle involved in frowning) while inhibiting the activation of the zygomaticas major. We found that when participants held the pen between their teeth, they perceived happiness to persist longer when the expression changed from happy to neutral, and perceived happiness sooner when the expression changed from neutral to happy. Conversely, when they held the pen with their lips, participants saw sadness to persist longer when the expression changed from sad to neutral, and perceived sadness sooner when neutral faces turned into sad expressions. This first main result of our study replicates and extends the findings of Niedenthal *et al.* (2000 [5]), suggesting that facial feedback assists in emotion perception by helping to detect small changes in the intensity of an emotion that is congruent with the one expressed on one’s own face.

Interestingly, however, our facial feedback manipulation had no significant effect on the ability to detect a qualitative change of emotion: In the morph sequences where a happy expression changed to sad or a sad expression turned happy, the pen manipulation did not selectively facilitate or suppress emotion perception relative to a no-pen baseline. This outcome differs from the results of Niedenthal *et al.*’s (2001 [4]) study (Experiment 2) which found a general suppression effect on the perception of categorical changes between emotions with an unspecific pen manipulation. Not only did we fail to find a general suppression effect (which would have been apparent in later change detection in both pen conditions compared to the no pen condition), we also failed to find a congruency effect when the expression on the stimulus face was equivalent to the expression the participant was forced to hold: when participants were forced to smile, they neither detected happiness significantly sooner nor did they perceive happiness to persist significantly longer in the morph sequences. According to embodied cognition theory, we would have expected that activation of facial muscles that are specific for a certain emotional expression should facilitate the perception of this same emotion when seen on somebody else’s face. Alternatively, according to the findings of Niedenthal *et al.* (2001), we would have predicted the offset of congruent emotions to appear earlier, since the new emotion would be incompatible with the emotion expressed on one’s own face and hence participants should be more sensitive to changes. Interestingly, neither of these predictions held true. Instead, our findings suggest that facial feedback neither improves nor hampers emotion recognition when one emotion changes qualitatively into another.

Why should facial feedback only influence the perception of emotional intensity but not qualitative changes of facial expressions? One explanation might be that by morphing between different expressions (as done in the qualitative change conditions) we may have produced intermediate facial constructions that would not naturally occur. Morphing between two expressions might not manipulate facial expressions in the same way as muscular action does, whereas creating quantitative morphs may more closely resemble naturally occurring muscular action. In the absence of evidence distinguishing the effectiveness of morphed *vs.* natural transitions between facial expressions, this explanation awaits further empirical testing. An alternative explanation may be that we used the same number of images to capture qualitative transitions between different emotional states and quantitative transitions from an emotional to a neutral state. Thus, the former situation contained less uncertainty than the latter, where changes per frame were more subtle. Given the larger uncertainty associated with quantitative compared to qualitative changes, participants might have recurred to facial feedback as an additional source of evidence when merely the intensity of expression changed.

Why could we not replicate the findings of Niedenthal *et al*. (2001) in the present study? We note that several differences in methodology between the two studies may be responsible for the conflicting findings. First, Niedenthal *et al.* (2001) used 100 step morph sequences, while our sequences contained only 40 steps. Using less than half the amount of steps may have reduced the spread of our participants’ answers, and thus may have been responsible for the missing effects. Secondly, the morph sequences used by Niedenthal *et al.* (2001) were played continuously and participants were instructed to stop the movie as soon as they detected the change in emotion expression. In contrast, the morph sequences used in our study were self-paced (*i.e.*, participants manually proceeded to the next frame by button press until they detected a change in emotion expression). This implies that the morph sequences in our study appeared less dynamic than the sequences in the study by Niedenthal *et al*. (2001). These substantial differences in the methodology used render a direct comparison between the two studies difficult. The two experimental conditions (qualitative *vs.* quantitative change) make most sense when compared against each other, rather than with the ones employed by Niedenthal *et al.* (2001). So, while methodological differences may account for the incompatible results between our and Niedenthal *et al.*’s (2001) study, these differences do not take away from our main finding, namely that facial feedback affects the perceived intensity more that the quality of emotional expressions.

Whereas numerous studies have reported that women usually outperform men in emotion recognition tasks e.g., [17,18,19,20], we found no indication for a female advantage. This is interesting, since a recent study suggested that such gender differences are especially pronounced when the facial expressions are subtle [19]. Given that our task involved the detection of subtle changes in facial expression, we could have expected that women would outperform men also in the present study. It will be the aim of future research to further scrutinize the role of participant sex in emotion processing, specifically in the context of emotional change detection.

We found a congruency effect when morph sequences started or ended with neutral expressions. That is, participants saw happiness longer and sooner when smiling. Similarly, they perceived sadness longer and sooner while frowning. An interesting question for future research will be whether the intensity of the induced expression on one’s own face modulates the congruency effect found in the present study.

## 5. Conclusion

Taken together, our results indicate that the activation/suppression technique developed by Strack *et al.* [13] modulates the detection of onset or offset of a single facial emotion but affects the detection of a change from one facial emotion to another to a lesser extent. These results are of theoretical importance in the field of embodied cognition and more specifically for the facial feedback theory because our findings suggest that facial feedback is indeed useful when interpreting other people’s facial expressions, especially when we have to evaluate the intensity of a facial expression.
